# Autophagy-related gene *P4HB*: a novel diagnosis and prognosis marker for kidney renal clear cell carcinoma

**DOI:** 10.18632/aging.102715

**Published:** 2020-01-30

**Authors:** Longxiang Xie, Huimin Li, Lu Zhang, Xiaoyu Ma, Yifang Dang, Jinshuai Guo, Jiahao Liu, Linna Ge, Fangmei Nan, Huan Dong, Zhongyi Yan, Xiangqian Guo

**Affiliations:** 1 Department of Predictive Medicine, Institute of Biomedical Informatics, Cell Signal Transduction Laboratory, Bioinformatics Center, Henan Provincial Engineering Center for Tumor Molecular Medicine, School of Basic Medical Sciences, Henan University, Kaifeng 475004, China

**Keywords:** autophagy, P4HB, KIRC, diagnostic biomarker, prognostic biomarker

## Abstract

Autophagy can protect cells and organisms from stressors such as nutrient deprivation, and is involved in many pathological processes including human cancer. Therefore, it is necessary to investigate the role of autophagy-related genes (ARGs) in cancer. In this study, we investigated the gene expression of 222 ARGs in 1048 Kidney Renal Clear Cell Carcinoma (KIRC) cases, from 5 independent cohorts. The gene expression of ARGs were first evaluated in the The Cancer Genome Atlas (TCGA) by Recevier Operating Characteristic (ROC) analysis to select potential biomarkers with extremely high ability in KIRC detection (AUC≥0.85 and *p*<0.0001). Then *in silico* procedure progressively leads to the selection of two genes in a three rounds of validation performed in four human KIRC-patients datasets including two independent Gene Expression Omnibus (GEO) datasets, Oncomine dataset and Human Protein Atlas dataset. Finally, only *P4HB* (Prolyl 4-hydroxylase, beta polypeptide) gene was experimentally validated by RT-PCR between control kidney cells and cancer cells. Following univariate and multivariate analyses of TCGA-KIRC clinical data showed that *P4HB* expression is an independent prognostic indicator of unfavorable overall survival (OS) for KIRC patients. Based on these findings, we proposed that *P4HB* might be one potential novel KIRC diagnostic and prognostic biomarker at both mRNA and protein levels.

## INTRODUCTION

Renal cell carcinoma (RCC), one of the most common malignancies of the urinary system, constitutes 3% of malignant tumors in adult [1]. The most common pathological type is Kidney Renal Clear Cell Carcinoma (KIRC), which is associated with high morbidity and poor prognosis [2]. Clinicopathological risk factors cannot sufficiently identify KIRC patients with a high risk of disease progression [3]. Currently, molecular biomarkers have been shown to guide the diagnosis, prognosis and therapy for KIRC patients. For example, IL13RA2 has been reported to involve the acquired sunitinib-resistance in KIRC [4]. High *SK1* expression can increase the invasion and angiogenesis abilities of cancer cell in a autocrine and paracrine manner respectively, and lead to a shorter survival in KIRC [5]. However, these reported potential biomarkers and functional important genes have not been tested in larger clinical cohorts. The stability and effectiveness of these biomarkers for KIRC diagnosis, prognosis and treatment response remain to be confirmed.

Autophagy is an intricate and critical homeostatic process in eukaryotic cells [6]. When stimulated by starvation and hypoxia, defective organelles are separated from cytoplasm and encircled by autophagosome (a double-membrane vesicle) [7]. Autophagosomes could mature by fusing with lysosomes to become autolysosomes which contain hydrolases to degrade the components encircled [8–10]. Depending on the conditions, autophagy has both protective and harmful effects including pro- or anti-tumor effects. For instance, chaperone-mediated autophagy degrades different types of substrates, with both cancer-suppressor and cancer-promoting activity [11]. The genes involved in the process of autophagy are called autophagy-related genes (ARGs) [12, 13]. Recent study has reported that ARGs can act as potential therapeutic targets to regulate epithelial to mesenchymal transition (EMT) in renal cell carcinoma [14]. In the present study, we explored the expression variations of 222 ARG genes in KIRC and investigated the potency as biomarkers in KIRC by analyzing 5 independent public datasets, and finally identified P4HB as a novel KIRC diagnosis and prognosis biomarker.

## RESULTS

**Figure 1:**
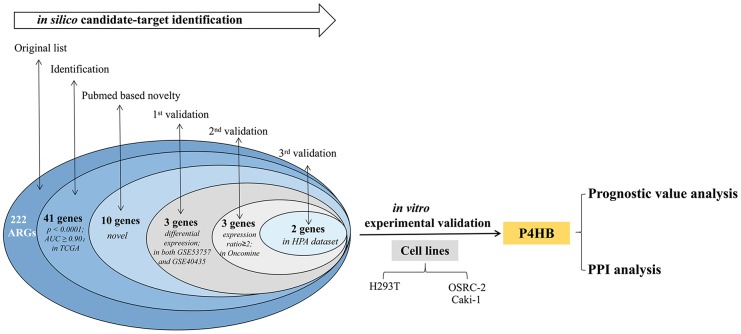
**Procedure for the selection and validation of the diagnosis and prognosis biomarkers in KIRC.**

The overall flowchart of this study is shown in Figure 1.

### Selection phase of ARGs in the TCGA-KIRC

The expression level of each of the 222 ARGs reported in Supplementary Table 1 was compared between KIRC and normal kidney in TCGA-KIRC dataset, which contains 533 KIRC biopsies and 72 normal kidney biopsies, and 177 above ARG members (80%) show the significant differential expression (*p*<0.05) between KIRC and normal kidney (Supplementary Table 2). For each of 222 genes, the computed AUC from ROC analysis was shown. Expression of 41 ARG genes was found to more effectively discriminate KIRC from normal kidney with AUC ≥ 0.9 and *p* < 0.0001 (Table 1). By NCBI Pubmed search, we found that 31 of these 41 ARG genes have been reported to be involved in KIRC, while remaining 10 genes have never been demonstrated to be directly related to KIRC (performed on April 1, 2019). Such 10 genes (*GABARAPL1*, *P4HB*, *ATG12*, *RAB24*, *CASP4*, *VAMP7*, *NLRC4*, *NRG3*, *PEX3* and *EEF2K*) are here considered as novel KIRC biomarker candidates.

**Table 1:** The 41 ARGs selected based on TCGA data and their following validation in other independent datasets.

**No.**	**Gene symbol**	**Screening phase (in TCGA-KIRC dataset)**			**Novelty (in PubMed)**	**First round validation (in the two GEO datasets)**	**Second round validation (in the Beroukhim dataset, Oncomine)**	**Third round validation (in the HPA dataset)**	**RT-PCR experiment validation**	**Full validation**
		**605 patients**				**346 patients**	**38 patients**	**59 patients**	**Control and kidney cancer cells**	
		**t test *p* value KIRC vs Normal**	**Ratio KIRC/Normal**	**AUC**	**ARG reported for KIRC in PubMed**	**Validation result**	**Validation result**	**Validation result**	**RT-PCR**	
1	*CDKN2A*	<0.0001	3.3554	0.9894	1	-	-	-	-	-
2	*CXCR4*	<0.0001	1.3376	0.9771	1	-	-	-	-	-
3	*MTOR*	<0.0001	0.8954	0.9757	1	-	-	-	-	-
4	*GAPDH*	<0.0001	1.0954	0.9688	1	-	-	-	-	-
5	*ERBB2*	<0.0001	0.8791	0.9653	1	-	-	-	-	-
6	*VEGFA*	<0.0001	1.2856	0.9643	1	-	-	-	-	-
7	*RGS19*	<0.0001	1.28	0.9609	1	-	-	-	-	-
8	*GNB2L1*	<0.0001	1.0845	0.9551	1	-	-	-	-	-
9	*BAX*	<0.0001	1.134	0.9497	1	-	-	-	-	-
10	*BID*	<0.0001	1.1824	0.9496	1	-	-	-	-	-
11	*C17orf88*	<0.0001	0.1622	0.9492	1	-	-	-	-	-
12	*HSPB8*	<0.0001	1.2442	0.9464	1	-	-	-	-	-
13	*EIF4EBP1*	<0.0001	1.234	0.9444	1	-	-	-	-	-
14	*BAG1*	<0.0001	0.8865	0.9436	1	-	-	-	-	-
15	*RAF1*	<0.0001	0.9379	0.9431	1	-	-	-	-	-
16	*CASP1*	<0.0001	1.2376	0.9374	1	-	-	-	-	-
17	*CAPN2*	<0.0001	0.9399	0.9372	1	-	-	-	-	-
18	*BIRC5*	<0.0001	1.9741	0.9337	1	-	-	-	-	-
19	*DIRAS3*	<0.0001	0.6646	0.9313	1	-	-	-	-	-
20	*ATG16L2*	<0.0001	1.3165	0.9281	1	-	-	-	-	-
21	*LAMP1*	<0.0001	0.9506	0.9273	1	-	-	-	-	-
22	*ATG9B*	<0.0001	2.0534	0.927	1	-	-	-	-	-
23	*ATF4*	<0.0001	1.0851	0.9256	1	-	-	-	-	-
24	*BNIP3*	<0.0001	1.1341	0.9205	1	-	-	-	-	-
25	*TP73*	<0.0001	2.9322	0.9197	1	-	-	-	-	-
26	*ATF6*	<0.0001	0.9345	0.9107	1	-	-	-	-	-
27	*ATG5*	<0.0001	0.9481	0.9096	1	-	-	-	-	-
28	*PRKAR1A*	<0.0001	0.9565	0.9094	1	-	-	-	-	-
29	*RAB5A*	<0.0001	0.9495	0.9068	1	-	-	-	-	-
30	*FAS*	<0.0001	1.1497	0.9039	1	-	-	-	-	-
31	*IFNG*	<0.0001	5.193	0.9038	1	-	-	-	-	-
32	*GABARAPL1*	<0.0001	0.8589	0.9603	0	Yes	Yes	Yes	No	-
33	*P4HB*	<0.0001	1.1007	0.9644	0	Yes	Yes	Yes	Yes	√
34	*ATG12*	<0.0001	1.1101	0.9556	0	No	-	-	-	-
35	*RAB24*	<0.0001	1.144	0.9497	0	No	-	-	-	-
36	*CASP4*	<0.0001	1.134	0.9413	0	Yes	Yes	No	-	-
37	*VAMP7*	<0.0001	0.9431	0.9398	0	No	-	-	-	-
38	*NLRC4*	<0.0001	1.4631	0.9211	0	No	-	-	-	-
39	*NRG3*	<0.0001	1.4895	0.9193	0	No	-	-	-	-
40	*PEX3*	<0.0001	0.914	0.9117	0	No	-	-	-	-
41	*EEF2K*	<0.0001	1.0887	0.9116	0	No	-	-	-	-

### First-round validation in the GEO datasets

The 10 genes shown in Table 1 were then tested in two independent GEO datasets (GSE40435 and GSE53757) using GEO2R to screen DEGs (Differential expression gene). 1128 genes presented identical expression trends in two datasets. Then, 3 genes (namely *CASP4*, *P4HB* and *GABARAPL1*) were obtained by overlapping 1128 DEGs and 10 ARG genes reported above.

### Second-round validation in the oncomine database

Three genes from first-round validation in GEO datasets were then analyzed in the fourth dataset, namely the Beroukhim dataset derived from Oncomine database. The three genes haven been shown to have an expression ratio in KIRC/controls > 2-fold (Supplementary Table 3). *CASP4* is over-expressed in kidney tumors, with fold change 2.697-fold to normal kidney. Similar to *CASP4*, up-regulation of *P4HB* was also found in KIRC (fold change = 2.012). A lower expression level of *GABARAPL1* was found in KIRC.

ARGs showing very high discriminating ability (AUC>0.90) in the TCGA dataset were searched in Pubmed to identify those never reported in KIRC, and then were validated in a first round validation in the two GEO dataset. Genes passing the first validation were then validated in the Beroukhim dataset. Genes passing the second validation were then validated in the HPA dataset. Genes passing screening phase and all four validations were regards as novel potential KIRC biomarkers. *P4HB* was selected according to this procedure. Empty cells indicate lack of validation. Genes showing 0 value in the “Novelty” column are genes without records for KIRC in Pubmed, whereas genes showing 1 value are genes with records.

### Third-round validation in the HPA

**Figure 2:**
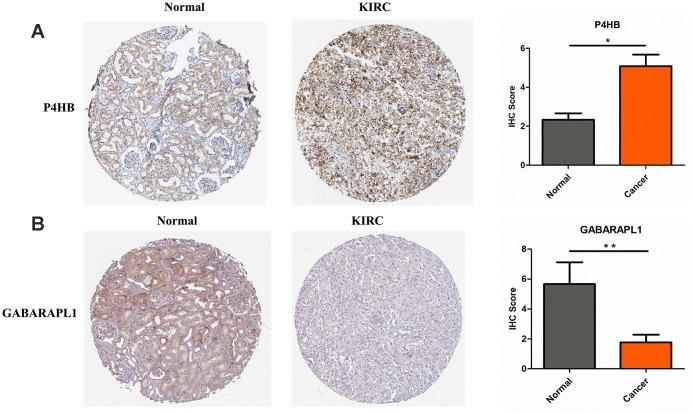
**P4HB protein expression was significantly higher in KIRC tissues in comparison with normal tissues, while GABRAPL1 protein expression was significantly lower.** Representive IHC images of P4HB (**A**) and GABRAPL1 (**B**) in normal (left) and KIRC (middle) tissues. Images were downloaded from HPA Database. Statistical analyses of the protein expression levels of P4HB and GABRAPL1 according to the information of normal and KIRC tissues (right). * *p*<0.05; ** *p*<0.01.

The protein expression levels of CASP4, P4HB and GABARAPL1 were then analyzed in HPA database. Fifty-nine histological section images for KIRC and normal kidney tissues were analyzed. The results showed that the protein expression levels of CASP4 have no significant difference between KIRCs and normal tissues (data not shown). Consistent to the above validation at mRNA level, the protein level of P4HB is significantly increased in KIRC tissues, and GABARAPL1 is decreased in KIRC tissues compared to that in normal tissues (Figure 2).

### Experimental validation

**Figure 3:**
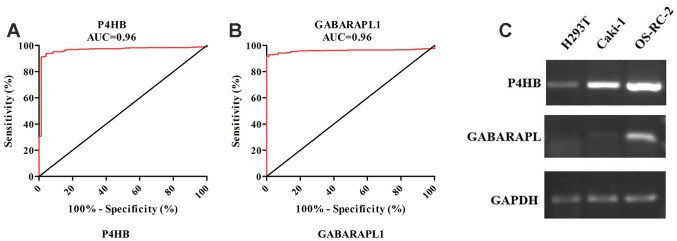
ROC analysis of *P4HB* and *GABRAPL1* never related to KIRC diagnosis showing a very high ability to discriminate controls from KIRC samples validated in TCGA (**A** and **B**). The AUC is plotted as sensitivity% vs 100-specifificity%. The calculated AUC is reported in each case. The *p* value is <0.0001 in all cases; Detection of *P4HB* and *GABARAPL1* mRNA expression level in H293T cell (normal kidney), OS-RC-2 and Caki-1 cell (cancer cells) by RT-PCR (**C**). *GAPDH* gene was used as the internal control. *P4HB* expression in cancer cells is clearly higher than normal kidney cell, while *GABARAPL1* expression in cancer cells is clearly lower than normal kidney cell.

According to the screening and validation steps as described above, the two genes P4HB and GABRAPL1 were considered as the novel candidates for KIRC biomarkers. Figure 3A and 3B showed the corresponding ROC curve of the two best candidates computed on the expression values reported in TCGA dataset. Both of them have AUC >0.9 and *p* < 0.0001, a very efficient ability to discriminate KIRCs from normal kidney.

The two candidates were further validated in cell lines and tissues by RT-PCR. In contrast with normal renal epithelial cell line 293 (H293T), the mRNA level of *P4HB* was elevated in KIRC cell lines OR-SC-2 and Caki-1 (Figure 3C). The mRNA level of *GABARAPL1* was also increased in KIRC cell line, which was inconsistent with above validation results. Hence, we just concentrated on *P4HB* for further study and analysis.

### Prognostic significance

**Figure 4:**
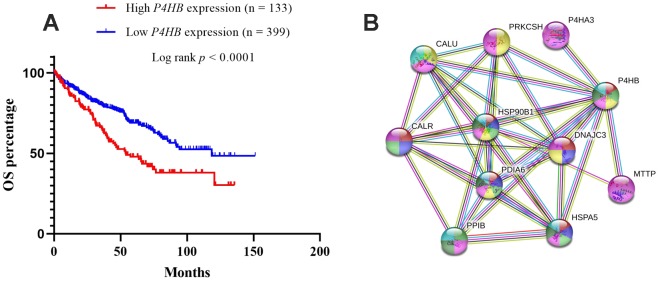
Kaplan-Meier survival analyses on differential *P4HB* expression groups with OS in the included 532 KIRC patients (**A**). The patients were stratified into high and low P4HB groups by quarter (25% upper vs 75% lower). Compared with low mRNA expression of *P4HB*, high *P4HB* expressions were significantly correlated with poor OS (*p* < 0.0001); The interaction network of P4HB protein with other proteins (**B**). CALR, HSP90B1, HSPA5, PPIB, MTTP, P4HA3, PDIA6, PRKCSH, and CALU physically/functionally connect P4HB. Note: The interaction network was obtained from STRING database.

The prognostic significance of *P4HB* transcription expression was investigated based on survival data in 532 TCGA-KIRC patients. Both quarter and median are typical options to stratify cancer patients into high and low gene expression groups. Patients were split into “high”- or “low”-expressing groups by the quarter or median of *P4HB* expression values. The results showed that high expression of *P4HB* mRNA was related to significantly worse OS for KIRC patients (*p*<0.0001) when using either the median or quarter as the cut-off (Figure 4A and Supplementary Figure 1). Consequently, we determined the independent prognostic value of *P4HB* in KIRC. The association between *P4HB* expression and the clinic-pathological parameters of KIRC patients is shown in Table 2. Results showed that the high *P4HB* expression group has a significantly higher ratio of patients in advanced stages (III/IV) (71/61 vs. 136/262; *p*<0.0001), recurrence status (9/20 vs. 15/101; *p*=0.026), and death (64/69 vs. 111/288; *p*<0.0001) compared to the low *P4HB* expression group.

**Table 2:** The association between *P4HB* expression and the demographic and clinicopathological parameters of patients with primary KIRC in the TCGA.

**Parameters**	***P4HB* mRNA expressio**				
		**High (n=133)**	**Low (n=399)**	***χ*^2^**	***p* value**
Age (Mean ± SD)		60.97±11.97	60.45±12.20		0.670
Gender	Female	37	149	3.979	0.058
	Male	96	250		
Clinical stage	I/II	61	262	16.026	<0.0001
	III/IV	71	136		
	Discrepancy	1	1		
Smoking history	1	10	35	1.000	0.056
	2/3/4/5	10	31		
	Null	113	333		
Recurrence status	No	20	101	5.505	0.026
	Yes	9	15		
	Null	104	283		
Living status	Living	69	288	18.623	<0.0001
	Dead	64	111		

To explore whether *P4HB* high expression could be an independent predictor for KIRC patients, univariate and multivariate Cox regression analyses were conducted. In univariate Cox regression analyses, the higher stage and high *P4HB* mRNA expression exhibited unfavorable effects on OS (*p*<0.0001 and *p*<0.0001, respectively) (Table 3). In multivariate analysis, the higher stage and higher *P4HB* mRNA expression were independent unfavorable biomarkers of OS (*p* < 0.0001 and *p*=0.007, respectively) (Table 3). These results strongly indicated that *P4HB* could be an independent unfavorable prognostic biomarker in KIRC.

**Table 3:** Univariate and multivariate analysis of OS in patients with primary KIRC

**Parameters OS**	**Univariate analysis**				**Multivariate analysis**			
	**HR**	**95%CI**		***p***	**HR**	**95%CI**		***p***
Age ≥60 vs <60	0.553	0.404	0.757	<0.0001	0.642	0.468	0.830	0.006
Female vs Male	1.060	0.779	1.442	0.710				
Clinical stage III/IV vs I/II	3.865	2.820	5.297	<0.0001	3.462	2.513	4.770	<0.0001
Smoking history 2/3/4/5 vs 1	0.778	0.254	2.383	0.661				
*P4HB* expression High vs Low	1.853	1.362	2.520	<0.0001	1.518	1.109	2.076	0.007

To study the potential significance of *P4HB* in KIRC, we used GSEA to compare the high expression and low expression of *P4HB* in the TCGA dataset. The result indicated that several vital regulatory genes involved in pentose phosphate pathway, fructose and mannose metabolism, amino sugar and nucleotide sugar metabolism, galactose metabolism, intestinal immune network for IGA production, proteasome, N-glycan biosynthesis were enriched in cells with high *P4HB* expression (Supplementary Figure 2).

### Interaction networks of P4HB

The STRING database was used to explore known and predicted protein–protein association with P4HB. As shown in Figure 4, the top 10 predicted functional partners were as follows: CALR (score = 0.999), HSP90B1 (score = 0.998), HSPA5 (score = 0.991), PPIB (score = 0.986), MTTP (score = 0.987), P4HA3 (score = 0.994), PDIA6 (score = 0.994), PRKCSH (score = 0.987), CALU (score = 0.991), and DNAJC3 (score = 0.986) (Figure 4B). Function enrichment analysis against gene ontology in this network indicated that for biological processes, this network is most enriched in response to endoplasmic reticulum (ER) stress, endoplasmic reticulum unfolded protein response, protein folding and post-translational protein modification, while for cellular components, it is significantly enriched in endoplasmic reticulum lumen, endoplasmic reticulum chaperone complex, and melanosome.

## DISCUSSION

Autophagy is important for sustaining cellular homeostasis through degrading and recycling organelles and proteins in eukaryotes [6]. This process can aid in the proliferation and survival of terminal cancers [6]. There is increasing evidence that targeting autophagy can promote the efficacy of many cancer therapies [15]. Recently, three ARGs including *WIPI1*, *BAG1* and *PEX3* were found to be melanoma diagnostic biomarkers with high AUC values, sensibility and specificity values [11]. Jonasch et al. had showed that autophagy possesses an anticancer role in KIRC tumorigenesis [16]. The monoallelic loss and/or mutation of autophagy-related gene *ATG7* was found to be frequently associated with KIRC, and its low expression correlated with KIRC progression [16]. This implies that ARGs may be considered as potential co-targets in the KIRC therapeutic strategies. Nevertheless, an extensive analysis of ARGs’ gene and protein expression levels in human KIRC samples has not been reported before.

The current study represents the first systemic analysis of the expression levels of ARGs in KIRC tissues. 222 ARGs’ expression were analyzed in 1048 human KIRC samples by transcriptomic and proteomic data. According to a multi-step selection and validation procedure (Figure 1), *P4HB* was predicted to be a novel ARG biomarker in KIRC samples, which shows high specificity and sensitivity values (Figure 3A). P4HB (Prolyl 4-hydroxylase, beta polypeptide), also known as protein disulfide isomerase, is a multifunctional protein that catalyzes the formation and rearrangement of disulfide bonds. It can act as a molecular chaperone to refine misfolded proteins in response to endoplasmic reticulum (ER) stress. *P4HB* is significantly increased in several solid tumors including bladder cancer [17, 18], brain and CNS cancer, lung cancer, ovarian cancer [19], prostate cancer [20, 21]. Moreover, *P4HB* is associated with temozolomide (TMZ) resistance in GBM cells [22]. Recently, Zhou et al. had shown that *P4HB* knockdown can induce the apoptosis of human HT29 colon cancer cell through generating reactive oxygen species and inhibiting STAT3 signaling [23]. Yusenko et al. and Higgins et al. had demonstrated that the transcriptional expression of *P4HB* in KIRC specimens is significantly higher than that in the non-tumor tissues (fold changes were 2.937 and 2.435, respectively) [24, 25]. The current study confirmed that *P4HB* is highly expressed in KIRC compared with that in the corresponding normal tissue, and is also increased in two KIRC cells (Figure 3C). All the results above show that *P4HB* might be a novel diagnosis KIRC marker. The prognostic value of *P4HB* expression in glioma and gastric cancer had been studied [26, 27], however, its prognostic value in other cancers including kidney cancer is still unknown. In 2018, Zhou et al. showed that diffuse glioma patients with high *P4HB* expression has a poor OS, and may function in tumor progression of diffuse gliomas [26]. Zhao et al. demonstrated *P4HB* overexpression is correlated with TNM staging and peritoneum cavity metastasis in gastric cancer, and patients with high-expression of *P4HB* had a shorter disease-free survival (DFS) than those with low-expression [27]. In this study, we firstly found that patients with high *P4HB* mRNA expression has shorter overall survival than that patients with low *P4HB* mRNA expression by univariate and multivariate analysis, indicating that *P4HB* may be an independent unfavorable prognostic biomarker for OS in KIRC patients.

The ER is a cellular organelle responsible for secreted and membrane protein folding. ER stress and cell death typically through apoptosis were triggered by cellular stressors, for example, low glucose, hypoxia and deregulation of calcium homeostasis [28, 29]. Autophagy was found to be induced for cell survival after ER stress in renal proximal tubular cells [30]. PPI analysis of P4HB based on STRING database gained 10 top proteins (including CALR, HSP90B1, HSPA5, PPIB, MTTP, P4HA3, PDIA6, PRKCSH, CALU, DNAJC3) which can interact with P4HB. Functional enrichment analysis of these interaction partners showed enrichment in the “response to ER stress” and “ER unfolded protein response”. This indicates that P4HB may interact with ER stress related proteins such as HSP90B1, HSPA5 and PDIA6 to regulate autophagy in KIRC. Moreover, Hsp90 was found to be involved in the autophagy via regulating diverse signaling pathways, such as toll-like receptor (TLR)-mediated autophagy, Ulk1-mediated mitophagy, and chaperone-mediated autophagy (CMA) [31]. Cerezo et al. had demonstrated that by inhibiting HSPA5 specifically by a new compound HA15, the autophagy and apoptosis was induced, and the unfolded protein response (UPR) was increased [32]. Bai et al. had shown that PDIA6 is overexpressed in non-small cell lung cancer cells (NSCLC) and its overexpression can inhibit cisplatin-induced cell apoptosis and autophagy via the MAP4K1/JNK/c-Jun signaling pathway [33]. Hence, we assume that *P4HB* might regulate autophagy through these above signaling pathways in KIRC. Further studies are required to solve these remaining questions.

## CONCLUSIONS

In summary, *P4HB* as an autophagy related gene is found to be significantly increased in KIRCs at both mRNA and protein levels, showing a high ability of diagnosis and prognosis. Therefore, the further study of the molecular mechanism of *P4HB* in tumorigenesis and progression of KIRC may offer additional opportunity of therapeutic target identification for KIRC patients.

## MATERIALS AND METHODS

222 ARGs (Autophagy-related genes) were collected from HADb (Human Autophagy Database, http://www.autophagy.lu/clustering/) at March 2019.

### ARGs expression in different datasets

Selection phase: Expression of 222 ARG genes listed in Supplementary Table 1 was evaluated in The Cancer Genome Atlas (TCGA)-KIRC dataset (UCSC Xena, https://xena.ucsc.edu/) [34]. This dataset contains the mRNA expression data of 605 samples (533 KIRCs and 72 normal kidneys). ROC (receiver operating characteristic) analysis, the frequently-used method for binary assessment, was then performed to assess the effectiveness of the transcriptional expression of any interesting gene to discriminate KIRC from healthy samples. The computed area under the curve (AUC) value ranging from 0.5 to 1.0 indicates the discrimination ability from 50 to 100%.

First-round validation: Genes showing different expression levels between KIRC and normal kidney from above analysis, were then searched in NCBI Pubmed for to look for their any significance in KIRC, at the date of April 1, 2019. Search in “ALL fields” was performed to decrease false negative results. Genes with no any significance in KIRC in Pubmed were kept for next steps of investigations in another two independent datasets (GSE40435 and GSE53757) to validate their expression distinction between KIRC and normal kidney by using GEO2R. The gene expression profiling datasets (GSE40435 and GSE53757) were obtained from Gene Expression Omnibus (GEO, https://www.ncbi.nlm.nih.gov/gds). 101 pairs of KIRC and normal kidney specimens were enrolled in GSE40435 (platform: GPL10588 Illumina HumanHT-12 V4.0 expression beadchip) while 72 pairs of KIRC and normal ones were enrolled in GSE53757 (platform: GPL570 Affymetrix Human Genome U133 Plus 2.0 Array). Finally, three ARGs were found to be differentially expressed between KIRCs and normal kidneys in all the three cohorts of TCGA, GSE40435 and GSE53757.

Second-round validation: The three genes validated within the previous phases was verified using the Oncomine database (https://www.oncomine.org/resource/main.html) [35]. The Oncomine applies a combination of threshold values, namely, *p* value, fold change vs controls, and gene rank. Very strict thresholds were applied, namely, *p*≤0.0001, fold change ≥2, and gene rank top 10%.

Third-round validation: The 2 genes identified above step were then analyzed at the protein expression level in HPA (Human Protein Atlas, https://www.proteinatlas.org/) [36]. 48 KIRC tissues and 11 healthy kidney controls were retrieved. The IHC staining intensity in HPA database was scored from 0 to 2 (0, no staining; 1, weak staining; 2 strong staining). The staining extent was scored from 0 to 4 based on the percentage of immune-reactive tumor cells (0%, 1–5%, 6–25%, 26–75%, 76–100%). A score ranging from 0 to 8 was calculated by multiplying the staining extent score with the staining intensity score, resulting in a negative (0–4) staining or a positive (6–8) staining for each example.

Experimental Validation: The mRNA transcriptional level of *P4HB* and *GABARAPL1* was assessed in normal renal epithelial cell 293 (H293T) and renal cancer cell (OS-RC-2 and Caki-1) stored in our lab. Total RNAs of these three cells were extracted with Trizol reagent (Thermo, MA, USA) following the manufacturer’s instructions. The purity and concentration of the RNA was detected by a NanoDrop 2000 spectrophotometer (NanoDrop Technologies, Thermo Fisher Scientific, USA). cDNA was obtained by using the kit (PrimeScript II 1^st^ Strand cDNA Synthesis Kit, TaKaRa). An equal amount of total RNA was used as a template for RT-PCR (Reverse transcription polymerase chain reaction) with random primers. The RT-PCR products were visualized using a 1% agarose gel. The sequences of primers used were as follows: *P4HB* (Forward: 5-AGGCTGATGACATCGTGAACT-3; Reverse: 5-GGTATTTGGAGAACACGTCACTG-3); *GABARAPL1* (Forward: 5-ATGAAGTTCCAGTACAAGGAGGA-3; Reverse: 5-GCTTTTGGAGCCTTCTCTACAAT-3); *GAPDH* (Forward: 5-ATGACAACTTTGGTATCGTGG-3; Reverse: 5-AGGGATGATGTTCTGGAGAG-3).

### Protein-protein interactions (PPI) network analysis

STRING is a database of predicted functional interactions between proteins [37]. The STRING (https://string-db.org/) was carried out to obtain the functional protein–protein interactions (PPIs) of P4HB protein.

### Gene set enrichment analysis (GSEA)

GSEA software was used to explore the subtype specific gene expression patterns and potential cellular pathways. By using TCGA_KIRC dataset, the high group and low group were divided based on the average of mRNA expression of *P4HB* because the mRNA expression obeys the normal distribution for its large sample. Nominal *p* < 0.05 and a false discovery rate (FDR) < 25% had considered to be significantly enriched for enriched gene sets analysis.

### Statistical analysis

Statistical analysis was carried out using SPSS ver. 18 (SPSS Inc., Chicago, IL, USA). Chi-square test was used to assess the possible association between *P4HB* expression and clinic-pathological factors. Kaplan-Meier curves of OS were constructed in GraphPad Prism 8.0 by setting the quarter (upper 25% vs lower 75%) or median (upper 50% vs lower 50%) of *P4HB* expression as the cut-off, respectively. A log-rank test was performed to examine the significant differences between the low expression group and high expression group. Univariate and multivariate Cox regression models were performed to analyze the prognostic value of *P4HB* mRNA expression in terms of OS for KIRC. Factors with prognostic significance in the univariate analysis were contained in the subsequent multivariate analysis. *P* < 0.05 was regarded as statistically significant.

## Supplementary Material

Supplementary Figures

Supplementary Table 1

Supplementary Table 2

Supplementary Table 3
