# Supplementary material for: Autophagy-related gene P4HB: a novel diagnosis and prognosis marker for kidney renal clear cell carcinoma
Source: Aging (Albany NY). 2020 Jan 30;12(2):1828–42. doi: 10.18632/aging.102715 (PMC7053637; doi:10.18632/aging.102715)
Supplement: Supplementary Figures [file aging-12-102715-s004..pdf]

SUPPLEMENTARY FIGURES

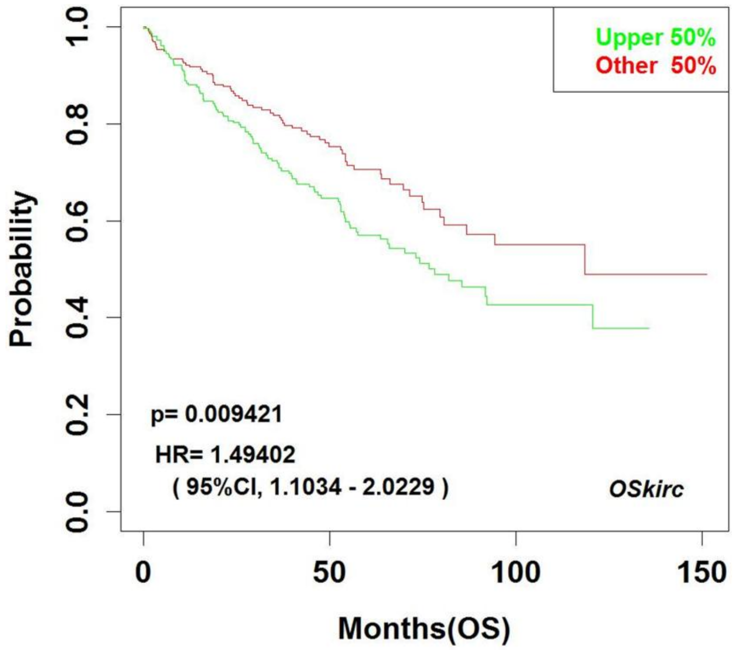

Supplementary Figure 1. Kaplan-Meier survival analyses on differential *P4HB* expression groups with OS in the included 532 KIRC patients. The patients were stratified into high and low *P4HB* groups by median (50% upper vs 50% lower). Compared with low mRNA expression of *P4HB*, high *P4HB* expressions were significantly correlated with poor OS ($p < 0.0001$);

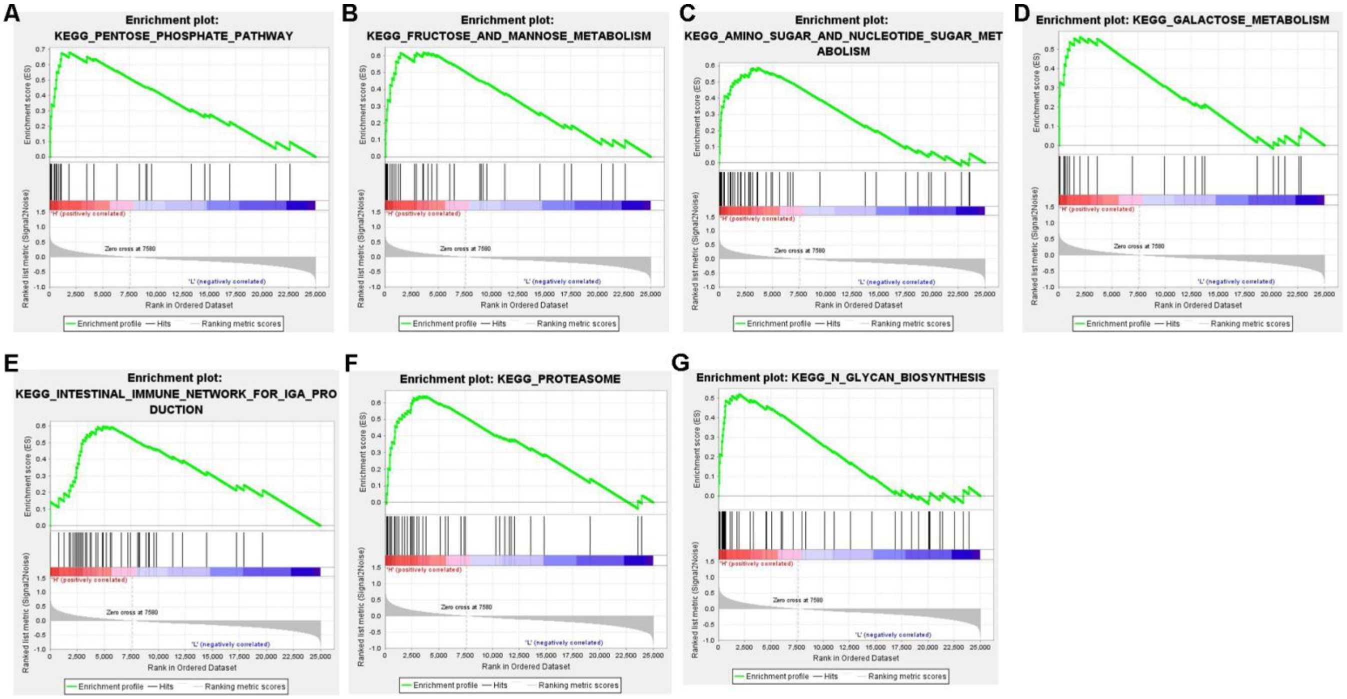

Supplementary Figure 2. Pathway involved in the pathogenesis of *P4HB* in TCGA-KIRC with GSEA. Enrichment curves are shown for activated gene sets related to pentose phosphate pathway (**A**), fructose and mannose metabolism(**B**), amino sugar and nucleotide sugar metabolism (**C**), galactose metabolism (**D**), intestinal immune network for IGA production (**E**), proteasome (**F**), N-glycan biosynthesis (**G**).
