# Supplementary material for: Autophagy-related gene P4HB: a novel diagnosis and prognosis marker for kidney renal clear cell carcinoma
Source: Aging (Albany NY). 2020 Jan 30;12(2):1828–42. doi: 10.18632/aging.102715 (PMC7053637; doi:10.18632/aging.102715)
Supplement: Supplementary Table 1 [file aging-12-102715-s003..docx]

**Supplementary Table 1. Autophagy Related genes (ARGs) investigated in this study.**

| **No** | **Gene symbol** | **Name** |
| --- | --- | --- |
| 1 | AMBRA1 | Autophagy/beclin-1 regulator 1 |
| 2 | APOL1 | Apolipoprotein L, 1 |
| 3 | ARNT | Aryl hydrocarbon receptor nuclear translocator |
| 4 | ARSA | Aarylsulfatase A |
| 5 | ARSB | Aarylsulfatase B |
| 6 | ATF4 | Aactivating transcription factor 4 |
| 7 | ATF6 | Activating transcription factor 6 |
| 8 | ATG10 | ATG10 autophagy related 10 homolog (S. cerevisiae) |
| 9 | ATG12 | ATG12 autophagy related 12 homolog (S. cerevisiae) |
| 10 | ATG16L1 | ATG16 autophagy related 16-like 1 (S. cerevisiae) |
| 11 | ATG16L2 | ATG16 autophagy related 16-like 2 (S. cerevisiae) |
| 12 | ATG2A | ATG2 autophagy related 2 homolog A (S. cerevisiae) |
| 13 | ATG2B | ATG2 autophagy related 2 homolog B (S. cerevisiae) |
| 14 | ATG3 | ATG3 autophagy related 3 homolog (S. cerevisiae) |
| 15 | ATG4A | ATG4 autophagy related 4 homolog A (S. cerevisiae) |
| 16 | ATG4B | ATG4 autophagy related 4 homolog B (S. cerevisiae) |
| 17 | ATG4C | ATG4 autophagy related 4 homolog C (S. cerevisiae) |
| 18 | ATG4D | ATG4 autophagy related 4 homolog D (S. cerevisiae) |
| 19 | ATG5 | ATG5 autophagy related 5 homolog (S. cerevisiae) |
| 20 | ATG7 | ATG7 autophagy related 7 homolog (S. cerevisiae) |
| 21 | ATG9A | ATG9 autophagy related 9 homolog A (S. cerevisiae) |
| 22 | ATG9B | ATG9 autophagy related 9 homolog B (S. cerevisiae) |
| 23 | ATIC | 5aminoimidazole4carboxamide ribonucleotide formyltransferase/IMP cyclohydrolase |
| 24 | BAG1 | BCL2-associated athanogene |
| 25 | BAG3 | BCL2-associated athanogene 3 |
| 26 | BAK1 | BCL2-antagonist/killer 1 |
| 27 | BAX | BCL2-associated X protein |
| 28 | BCL2 | B-cell CLL/lymphoma 2 |
| 29 | BCL2L1 | BCL2-like 1 |
| 30 | BECN1 | Beclin 1, autophagy related |
| 31 | BID | BH3 interacting domain death agonist |
| 32 | BIRC5 | baculoviral IAP repeat-containing 5 |
| 33 | BIRC6 | baculoviral IAP repeat-containing 6 |
| 34 | BNIP1 | BCL2/adenovirus E1B 19kDa interacting protein 1 |
| 35 | BNIP3 | BCL2/adenovirus E1B 19kDa interacting protein 3 |
| 36 | BNIP3L | BCL2/adenovirus E1B 19kDa interacting protein 3-like |
| 37 | C12orf44 | Chromosome 12 open reading frame 44 |
| 38 | C17orf88 | Chromosome 17 open reading frame 88 |
| 39 | CALCOCO2 | Calcium binding and coiled-coil domain 2 |
| 40 | CAMKK2 | Calcium/calmodulin-dependent protein kinase kinase 2, beta |
| 41 | CANX | Calnexin |
| 42 | CAPN1 | Calpain 1, (mu/I) large subunit |
| 43 | CAPN10 | Calpain 10 |
| 44 | CAPN2 | Calpain 2, (m/II) large subunit |
| 45 | CAPNS1 | Calpain, small subunit 1 |
| 46 | CASP1 | Caspase 1, apoptosis-related cysteine peptidase (interleukin 1, beta, convertase) |
| 47 | CASP3 | Caspase 3, apoptosis-related cysteine peptidase |
| 48 | CASP4 | Caspase 4, apoptosis-related cysteine peptidase |
| 49 | CASP8 | Caspase 8, apoptosis-related cysteine peptidase |
| 50 | CCL2 | Chemokine (C-C motif) ligand 2 |
| 51 | CCR2 | Chemokine (C-C motif) receptor 2 |
| 52 | CD46 | CD46 molecule, complement regulatory protein |
| 53 | CDKN1A | Cyclin-dependent kinase inhibitor 1A (p21, Cip1) |
| 54 | CDKN1B | Cyclin-dependent kinase inhibitor 1B (p27, Kip1) |
| 55 | CDKN2A | Cyclin-dependent kinase inhibitor 2A (melanoma, p16, inhibits CDK4) |
| 56 | CFLAR | CASP8 and FADD-like apoptosis regulator |
| 57 | CHMP2B | Chromatin modifying protein 2B |
| 58 | CHMP4B | Chromatin modifying protein 4B |
| 59 | CLN3 | Ceroid-lipofuscinosis, neuronal 3 |
| 60 | CTSB | Cathepsin B |
| 61 | CTSD | Cathepsin D |
| 62 | CTSL1 | Cathepsin L1 |
| 63 | CX3CL1 | Chemokine (C-X3-C motif) ligand 1 |
| 64 | CXCR4 | Chemokine (C-X-C motif) receptor 4 |
| 65 | DAPK1 | Death-associated protein kinase 1 |
| 66 | DAPK2 | Death-associated protein kinase 2 |
| 67 | DDIT3 | DNA-damage-inducible transcript 3 |
| 68 | DIRAS3 | DIRAS family, GTP-binding RAS-like 3 |
| 69 | DLC1 | Deleted in liver cancer 1 |
| 70 | DNAJB1 | DnaJ (Hsp40) homolog, subfamily B, member 1 |
| 71 | DNAJB9 | DnaJ (Hsp40) homolog, subfamily B, member 9 |
| 72 | DRAM1 | DNA-damage regulated autophagy modulator 1 |
| 73 | EDEM1 | ER degradation enhancer, mannosidase alpha-like 1 |
| 74 | EEF2 | Eukaryotic translation elongation factor 2 |
| 75 | EEF2K | Eukaryotic elongation factor-2 kinase |
| 76 | EGFR | Epidermal growth factor receptor |
| 77 | EIF2AK2 | Eukaryotic translation initiation factor 2-alpha kinase 2 |
| 78 | EIF2AK3 | Eukaryotic translation initiation factor 2-alpha kinase 3 |
| 79 | EIF2S1 | Eukaryotic translation initiation factor 2, subunit 1 alpha, 35kDa |
| 80 | EIF4EBP1 | Eukaryotic translation initiation factor 4E binding protein 1 |
| 81 | EIF4G1 | Eeukaryotic translation initiation factor 4 gamma, 1 |
| 82 | ERBB2 | v-Erb-b2 erythroblastic leukemia viral oncogene homolog 2, neuro/glioblastoma derived oncogene homolog (avian) |
| 83 | ERN1 | Endoplasmic reticulum to nucleus signaling 1 |
| 84 | ERO1L | ERO1-like (S. cerevisiae) |
| 85 | FADD | Fas (TNFRSF6)-associated via death domain |
| 86 | FAM48A | Family with sequence similarity 48, member A |
| 87 | FAS | Fas (TNF receptor superfamily, member 6) |
| 88 | FKBP1A | FK506 binding protein 1A, 12kDa |
| 89 | FKBP1B | FK506 binding protein 1B, 12.6 kDa |
| 90 | FOS | FBJ murine osteosarcoma viral oncogene homolog |
| 91 | FOXO1 | Forkhead box O1 |
| 92 | FOXO3 | Forkhead box O3 |
| 93 | GAA | Glucosidase, alpha; acid |
| 94 | GABARAP | GABA(A) receptor-associated protein |
| 95 | GABARAPL1 | GABA(A) receptor-associated protein like 1 |
| 96 | GABARAPL2 | GABA(A) receptor-associated protein-like 2 |
| 97 | GAPDH | Glyceraldehyde-3-phosphate dehydrogenase |
| 98 | GNAI3 | Guanine nucleotide binding protein (G protein) alpha inhibiting activity polypeptide 3 |
| 99 | GNB2L1 | Guanine nucleotide binding protein (G protein), beta polypeptide 2-like 1 |
| 100 | GOPC | Golgi-associated PDZ and coiled-coil motif containing |
| 101 | GRID1 | Glutamate receptor, ionotropic, delta 1 |
| 102 | GRID2 | Glutamate receptor, ionotropic, delta 2 |
| 103 | HDAC1 | Histone deacetylase 1 |
| 104 | HDAC6 | Histone deacetylase 6 |
| 105 | HGS | Hepatocyte growth factor-regulated tyrosine kinase substrate |
| 106 | HIF1A | Hypoxia inducible factor 1, alpha subunit (basic helix-loop-helix transcription factor) |
| 107 | HSP90AB1 | Heat shock protein 90kDa alpha (cytosolic), class B member 1 |
| 108 | HSPA5 | Heat shock 70kDa protein 5 (glucose-regulated protein, 78kDa) |
| 109 | HSPA8 | Heat shock 70kDa protein 8 |
| 110 | HSPB8 | Heat shock 22kDa protein 8 |
| 111 | IFNG | Interferon, gamma |
| 112 | IKBKB | Inhibitor of kappa light polypeptide gene enhancer in B-cells, kinase beta |
| 113 | IKBKE | Inhibitor of kappa light polypeptide gene enhancer in B-cells, kinase epsilon |
| 114 | IL24 | Interleukin 24 |
| 115 | IRGM | Immunity-related GTPase family, M |
| 116 | ITGA3 | Integrin, alpha 3 (antigen CD49C, alpha 3 subunit of VLA-3 receptor) |
| 117 | ITGA6 | Integrin, alpha 6 |
| 118 | ITGB1 | Integrin, beta 1 (fibronectin receptor, beta polypeptide, antigen CD29 includes MDF2, MSK12) |
| 119 | ITGB4 | Integrin, beta 4 |
| 120 | ITPR1 | Inositol 1,4,5-triphosphate receptor, type 1 |
| 121 | KIAA0226 | KIAA0226 |
| 122 | KIAA0652 | KIAA0652 |
| 123 | KIAA0831 | KIAA0831 |
| 124 | KIF5B | Kinesin family member 5B |
| 125 | KLHL24 | Kelch-like 24 (Drosophila) |
| 126 | LAMP1 | Lysosomal-associated membrane protein 1 |
| 127 | LAMP2 | Lysosomal-associated membrane protein 2 |
| 128 | MAP1LC3A | Microtubule-associated protein 1 light chain 3 alpha |
| 128 | MAP1LC3B | Microtubule-associated protein 1 light chain 3 beta |
| 130 | MAP1LC3C | Microtubule-associated protein 1 light chain 3 gamma |
| 131 | MAP2K7 | Mitogen-activated protein kinase kinase 7 |
| 132 | MAPK1 | Mitogen-activated protein kinase 1 |
| 133 | MAPK3 | Mitogen-activated protein kinase 3 |
| 134 | MAPK8 | Mitogen-activated protein kinase 8 |
| 135 | MAPK8IP1 | Mitogen-activated protein kinase 8 interacting protein 1 |
| 136 | MAPK9 | Mitogen-activated protein kinase 9 |
| 137 | MBTPS2 | Membrane-bound transcription factor peptidase, site 2 |
| 138 | MLST8 | MTOR associated protein, LST8 homolog (S. cerevisiae) |
| 139 | MTMR14 | Myotubularin related protein 14 |
| 140 | MTOR | Mechanistic target of rapamycin (serine/threonine kinase) |
| 141 | MYC | v-myc myelocytomatosis viral oncogene homolog (avian) |
| 142 | NAF1 | Nuclear assembly factor 1 homolog (S. cerevisiae) |
| 143 | NAMPT | Nicotinamide phosphoribosyltransferase |
| 144 | NBR1 | Neighbor of BRCA1 gene 1 |
| 145 | NCKAP1 | NCK-associated protein 1 |
| 146 | NFE2L2 | Nuclear factor (erythroid-derived 2)-like 2 |
| 147 | NFKB1 | Nuclear factor of k light polypeptide gene enhancer in B-cells 1 |
| 148 | NKX2-3 | NK2 transcription factor related, locus 3 (Drosophila) |
| 149 | NLRC4 | NLR family, CARD domain containing 4 |
| 150 | NPC1 | Niemann-Pick disease, type C1 |
| 151 | NRG1 | Neuregulin 1 |
| 152 | NRG2 | Neuregulin 2 |
| 153 | NRG3 | Neuregulin 3 |
| 154 | P4HB | Prolyl 4-hydroxylase, beta polypeptide |
| 155 | PARK2 | Parkinson disease (autosomal recessive, juvenile) 2, parkin |
| 156 | PARP1 | Poly (ADP-ribose) polymerase 1 |
| 157 | PEA15 | Phosphoprotein enriched in astrocytes 15 |
| 158 | PELP1 | Proline, glutamate and leucine rich protein 1 |
| 159 | PEX14 | Peroxisomal biogenesis factor 14 |
| 160 | PEX3 | Peroxisomal biogenesis factor 3 |
| 161 | PIK3C3 | Phosphoinositide-3-kinase, class 3 |
| 162 | PIK3R4 | Phosphoinositide-3-kinase, regulatory subunit 4 |
| 163 | PINK1 | PTEN induced putative kinase 1 |
| 164 | PPP1R15A | Protein phosphatase 1, regulatory (inhibitor) subunit 15A |
| 165 | PRKAB1 | Protein kinase, AMP-activated, beta 1 non-catalytic subunit |
| 166 | PRKAR1A | Protein kinase, cAMP-dependent, regulatory, type I, alpha |
| 167 | PRKCD | Protein kinase C, delta |
| 168 | PRKCQ | Protein kinase C, theta |
| 169 | PTEN | Phosphatase and tensin homolog |
| 170 | PTK6 | PTK6 protein tyrosine kinase 6 |
| 171 | RAB11A | RAB11A, member RAS oncogene family |
| 172 | RAB1A | RAB1A, member RAS oncogene family |
| 173 | RAB24 | RAB24, member RAS oncogene family |
| 174 | RAB33B | RAB33B, member RAS oncogene family |
| 175 | RAB5A | RAB5A, member RAS oncogene family |
| 176 | RAB7A | RAB7A, member RAS oncogene family |
| 177 | RAC1 | Ras-related C3 botulinum toxin substrate 1 (rho family small GTP bind. protein Rac1) |
| 178 | RAF1 | v-Raf-1 murine leukemia viral oncogene homolog 1 |
| 179 | RB1 | Retinoblastoma 1 |
| 180 | RB1CC1 | RB1-inducible coiled-coil 1 |
| 181 | RELA | v-Rel reticuloendotheliosis viral oncogene homolog A (avian) |
| 182 | RGS19 | Regulator of G-protein signaling 19 |
| 183 | RHEB | Ras homolog enriched in brain |
| 184 | RPS6KB1 | Ribosomal protein S6 kinase, 70kDa, polypeptide 1 |
| 185 | RPTOR | Regulatory associated protein of MTOR, complex 1 |
| 186 | SAR1A | SAR1 homolog A (S. cerevisiae) |
| 187 | SERPINA1 | Serpin peptidase inhibitor, clade A (alpha-1 antiproteinase, antitrypsin), member 1 |
| 188 | SESN2 | Sestrin 2 |
| 189 | SH3GLB1 | SH3-domain GRB2-like endophilin B1 |
| 190 | SIRT1 | Sirtuin (silent mating type information regulation 2 homolog) 1 (S. cerevisiae) |
| 191 | SIRT2 | Sirtuin (silent mating type information regulation 2 homolog) 2 (S. cerevisiae) |
| 192 | SPHK1 | Sphingosine kinase 1 |
| 193 | SPNS1 | Spinster homolog 1 (Drosophila) |
| 194 | SQSTM1 | Sequestosome 1 |
| 195 | ST13 | Ssuppression of tumorigenicity 13 (colon carcinoma) |
| 196 | STK11 | Serine/threonine kinase 11 |
| 197 | TBK1 | TANK-binding kinase 1 |
| 198 | TM9SF1 | Transmembrane 9 superfamily member 1 |
| 199 | TMEM49 | Transmembrane protein 49 |
| 200 | TMEM74 | Transmembrane protein 74 |
| 201 | TNFSF10 | Tumor necrosis factor (ligand) superfamily, member 10 |
| 202 | TP53 | Tumor protein p53 |
| 203 | TP53INP2 | Tumor protein p53 inducible nuclear protein 2 |
| 204 | TP63 | Tumor protein p63 |
| 205 | TP73 | Tumor protein p73 |
| 206 | TSC1 | Tuberous sclerosis 1 |
| 207 | TSC2 | Tuberous sclerosis 2 |
| 208 | TUSC1 | Tumor suppressor candidate 1 |
| 209 | ULK1 | unc-51-like kinase 1 (C. elegans) |
| 210 | ULK2 | unc-51-like kinase 2 (C. elegans) |
| 211 | ULK3 | unc-51-like kinase 3 (C. elegans) |
| 212 | USP10 | Ubiquitin specific peptidase 10 |
| 213 | UVRAG | UV radiation resistance associated gene |
| 214 | VAMP3 | Vesicle-associated membrane protein 3 (cellubrevin) |
| 215 | VAMP7 | Vesicle-associated membrane protein 7 |
| 216 | VEGFA | Vascular endothelial growth factor A |
| 217 | WDFY3 | WD repeat and FYVE domain containing 3 |
| 218 | WDR45 | WD repeat domain 45 |
| 219 | WDR45L | WDR45-like |
| 220 | WIPI1 | WD repeat domain, phosphoinositide interacting 1 |
| 221 | WIPI2 | WD repeat domain, phosphoinositide interacting 2 |
| 222 | ZFYVE1 | Zinc finger, FYVE domain containing 1 |
