# Supplementary material for: Autophagy-related gene P4HB: a novel diagnosis and prognosis marker for kidney renal clear cell carcinoma
Source: Aging (Albany NY). 2020 Jan 30;12(2):1828–42. doi: 10.18632/aging.102715 (PMC7053637; doi:10.18632/aging.102715)
Supplement: Supplementary Table 2 [file aging-12-102715-s001..docx]

Supplementary Table 2. List of ARGs showing differential expression in KIRC vs healthy kidney (*p*<0.05), sorted according to the AUC value. The analysis was performed in TCGA dataset.

| No. | Gene  Name | *p*-value | Ratio KIRC  vs. Normal | | AUC | No. | Gene  Name | *p*-value | Ratio KIRC  vs. Normal | | AUC |
| --- | --- | --- | --- | --- | --- | --- | --- | --- | --- | --- | --- |
| 1 | AMBRA1 | <0.0001 | 0.9827 | ↓ | 0.7071 | 90 | ITGB4 | 0.0219 | 1.0359 | ↑ | 0.6150 |
| 2 | APOL1 | <0.0001 | 1.1431 | ↑ | 0.8816 | 91 | ITPR1 | <0.0001 | 0.9109 | ↓ | 0.8561 |
| 3 | ARNT | 0.0082 | 0.9885 | ↓ | 0.6020 | 92 | KIAA0226 | <0.0001 | 1.0292 | ↑ | 0.7173 |
| 4 | ARSA | <0.0001 | 1.0751 | ↑ | 0.8016 | 93 | KIAA0652 | 0.0010 | 0.9890 | ↓ | 0.6673 |
| 5 | ARSB | 0.0003 | 1.0368 | ↑ | 0.9407 | 94 | KIF5B | <0.0001 | 0.9609 | ↓ | 0.8583 |
| 6 | ATF4 | <0.0001 | 1.0851 | ↑ | 0.9256 | 95 | KLHL24 | 0.0177 | 0.9870 | ↓ | 0.6084 |
| 7 | ATF6 | <0.0001 | 0.9345 | ↓ | 0.9107 | 96 | LAMP1 | <0.0001 | 0.9506 | ↓ | 0.9273 |
| 8 | ATG12 | <0.0001 | 1.1101 | ↑ | 0.9556 | 97 | MAP1LC3A | <0.0001 | 0.9433 | ↓ | 0.7308 |
| 9 | ATG16L1 | <0.0001 | 1.0843 | ↑ | 0.8511 | 98 | MAP1LC3C | 0.0011 | 1.2060 | ↑ | 0.6189 |
| 10 | ATG16L2 | <0.0001 | 1.3165 | ↑ | 0.9281 | 99 | MAP2K7 | <0.0001 | 1.0367 | ↑ | 0.8043 |
| 11 | ATG2A | <0.0001 | 0.9681 | ↓ | 0.7442 | 100 | MAPK3 | <0.0001 | 1.0305 | ↑ | 0.7800 |
| 12 | ATG2B | <0.0001 | 0.9495 | ↓ | 0.8072 | 101 | MAPK8 | <0.0001 | 0.9160 | ↓ | 0.8561 |
| 13 | ATG4A | <0.0001 | 0.9767 | ↓ | 0.7070 | 102 | MAPK8IP1 | <0.0001 | 0.8870 | ↓ | 0.8506 |
| 14 | ATG4D | 0.0002 | 0.9624 | ↓ | 0.7069 | 103 | MAPK9 | 0.0004 | 1.0160 | ↑ | 0.6600 |
| 15 | ATG5 | <0.0001 | 0.9481 | ↓ | 0.9096 | 104 | MLST8 | 0.0095 | 1.0200 | ↑ | 0.6102 |
| 16 | ATG7 | 0.0496 | 1.0097 | ↑ | 0.5857 | 105 | MTMR14 | <0.0001 | 0.9583 | ↓ | 0.8318 |
| 17 | ATG9B | <0.0001 | 2.0534 | ↑ | 0.9270 | 106 | MTOR | <0.0001 | 0.8954 | ↓ | 0.9757 |
| 18 | BAG1 | <0.0001 | 0.8865 | ↓ | 0.9436 | 107 | MYC | <0.0001 | 1.1914 | ↑ | 0.8683 |
| 19 | BAG3 | <0.0001 | 0.9691 | ↓ | 0.7151 | 108 | NAF1 | 0.0008 | 0.9740 | ↓ | 0.6263 |
| 20 | BAX | <0.0001 | 1.1340 | ↑ | 0.9497 | 109 | NAMPT | 0.0002 | 1.0339 | ↑ | 0.6573 |
| 21 | BCL2 | <0.0001 | 1.0542 | ↑ | 0.7807 | 110 | NBR1 | <0.0001 | 0.9647 | ↓ | 0.8320 |
| 22 | BECN1 | <0.0001 | 0.9602 | ↓ | 0.8988 | 111 | NCKAP1 | <0.0001 | 0.9615 | ↓ | 0.8520 |
| 23 | BID | <0.0001 | 1.1824 | ↑ | 0.9496 | 112 | NFE2L2 | <0.0001 | 0.9583 | ↓ | 0.8422 |
| 24 | BIRC5 | <0.0001 | 1.9741 | ↑ | 0.9337 | 113 | NFKB1 | 0.0017 | 1.0185 | ↑ | 0.7017 |
| 25 | BIRC6 | <0.0001 | 0.9679 | ↓ | 0.6968 | 114 | NKX2-3 | <0.0001 | 3.2345 | ↑ | 0.6701 |
| 26 | BNIP3 | <0.0001 | 1.1341 | ↑ | 0.9205 | 115 | NLRC4 | <0.0001 | 1.4631 | ↑ | 0.9211 |
| 27 | BNIP3L | <0.0001 | 1.0761 | ↑ | 0.8944 | 116 | NPC1 | <0.0001 | 1.0675 | ↑ | 0.8394 |
| 28 | C17orf88 | <0.0001 | 0.1622 | ↓ | 0.9492 | 117 | NRG1 | 0.0011 | 0.9142 | ↓ | 0.6223 |
| 29 | CALCOCO2 | 0.0002 | 1.0133 | ↑ | 0.7016 | 118 | NRG3 | <0.0001 | 1.4895 | ↑ | 0.9193 |
| 30 | CAMKK2 | <0.0001 | 1.0561 | ↑ | 0.8978 | 119 | P4HB | <0.0001 | 1.1007 | ↑ | 0.9644 |
| 31 | CANX | <0.0001 | 1.0324 | ↑ | 0.7674 | 120 | PARK2 | <0.0001 | 0.8590 | ↓ | 0.8383 |
| 32 | CAPN1 | <0.0001 | 0.9790 | ↓ | 0.6154 | 121 | PARP1 | 0.0001 | 0.9795 | ↓ | 0.6973 |
| 33 | CAPN2 | <0.0001 | 0.9399 | ↓ | 0.9372 | 122 | PEA15 | <0.0001 | 1.0565 | ↑ | 0.8705 |
| 34 | CASP1 | <0.0001 | 1.2376 | ↑ | 0.9374 | 123 | PELP1 | 0.0009 | 1.0185 | ↑ | 0.6344 |
| 35 | CASP3 | <0.0001 | 1.0744 | ↑ | 0.8332 | 124 | PEX3 | <0.0001 | 0.9140 | ↓ | 0.9117 |
| 36 | CASP4 | <0.0001 | 1.1340 | ↑ | 0.9413 | 125 | PIK3C3 | 0.0010 | 0.9818 | ↓ | 0.6322 |
| 37 | CASP8 | <0.0001 | 1.0813 | ↑ | 0.8986 | 126 | PIK3R4 | <0.0001 | 0.9538 | ↓ | 0.8278 |
| 38 | CCR2 | <0.0001 | 1.2903 | ↑ | 0.7907 | 127 | PINK1 | <0.0001 | 0.9499 | ↓ | 0.8815 |
| 39 | CD46 | <0.0001 | 0.9547 | ↓ | 0.8783 | 128 | PPP1R15A | <0.0001 | 1.0651 | ↑ | 0.7329 |
| 40 | CDKN1A | <0.0001 | 1.0847 | ↑ | 0.7672 | 128 | PRKAB1 | <0.0001 | 0.9349 | ↓ | 0.8302 |
| 41 | CDKN1B | <0.0001 | 1.0263 | ↑ | 0.7276 | 130 | PRKAR1A | <0.0001 | 0.9565 | ↓ | 0.9094 |
| 42 | CDKN2A | <0.0001 | 3.3554 | ↑ | 0.9894 | 131 | PRKCD | <0.0001 | 0.9480 | ↓ | 0.8299 |
| 43 | CFLAR | <0.0001 | 1.0378 | ↑ | 0.7514 | 132 | PRKCQ | <0.0001 | 0.7876 | ↓ | 0.8936 |
| 44 | CHMP2B | <0.0001 | 0.9497 | ↓ | 0.8385 | 133 | PTK6 | 0.0010 | 1.1838 | ↑ | 0.6190 |
| 45 | CHMP4B | 0.0013 | 1.0132 | ↑ | 0.6207 | 134 | RAB11A | <0.0001 | 0.9796 | ↓ | 0.7255 |
| 46 | CTSB | <0.0001 | 1.0284 | ↑ | 0.6279 | 135 | RAB1A | <0.0001 | 0.9826 | ↓ | 0.7502 |
| 47 | CTSD | 0.0019 | 1.0209 | ↑ | 0.6249 | 136 | RAB24 | <0.0001 | 1.1440 | ↑ | 0.9497 |
| 48 | CTSL1 | 0.0011 | 1.0176 | ↑ | 0.6366 | 137 | RAB5A | <0.0001 | 0.9495 | ↓ | 0.9068 |
| 49 | CX3CL1 | <0.0001 | 1.0779 | ↑ | 0.8492 | 138 | RAB7A | <0.0001 | 0.9865 | ↓ | 0.7008 |
| 50 | CXCR4 | <0.0001 | 1.3376 | ↑ | 0.9771 | 139 | RAC1 | <0.0001 | 1.0194 | ↑ | 0.7163 |
| 51 | DAPK1 | 0.0001 | 0.9661 | ↓ | 0.6590 | 140 | RAF1 | <0.0001 | 0.9379 | ↓ | 0.9431 |
| 52 | DIRAS3 | <0.0001 | 0.6646 | ↓ | 0.9313 | 141 | RB1 | <0.0001 | 1.0281 | ↑ | 0.7372 |
| 53 | DLC1 | 0.0107 | 0.9835 | ↓ | 0.6172 | 142 | RB1CC1 | 0.0065 | 0.9856 | ↓ | 0.6389 |
| 54 | DNAJB9 | <0.0001 | 1.0508 | ↑ | 0.7171 | 143 | RELA | <0.0001 | 1.0330 | ↑ | 0.8426 |
| 55 | DRAM1 | <0.0001 | 1.0759 | ↑ | 0.8551 | 144 | RGS19 | <0.0001 | 1.2800 | ↑ | 0.9609 |
| 56 | EDEM1 | <0.0001 | 1.0225 | ↑ | 0.6802 | 145 | RPS6KB1 | <0.0001 | 1.0351 | ↑ | 0.8343 |
| 57 | EEF2 | <0.0001 | 1.0315 | ↑ | 0.8394 | 146 | RPTOR | 0.0200 | 1.0152 | ↑ | 0.6222 |
| 58 | EEF2K | <0.0001 | 1.0887 | ↑ | 0.9116 | 147 | SAR1A | 0.0021 | 0.9902 | ↓ | 0.6604 |
| 59 | EGFR | <0.0001 | 1.1026 | ↑ | 0.7975 | 148 | SERPINA1 | <0.0001 | 1.0906 | ↑ | 0.7721 |
| 60 | EIF4EBP1 | <0.0001 | 1.2340 | ↑ | 0.9444 | 149 | SESN2 | <0.0001 | 0.9489 | ↓ | 0.6798 |
| 61 | EIF4G1 | 0.0295 | 0.9905 | ↓ | 0.5925 | 150 | SH3GLB1 | <0.0001 | 1.0210 | ↑ | 0.7594 |
| 62 | ERBB2 | <0.0001 | 0.8791 | ↑ | 0.9653 | 151 | SIRT2 | <0.0001 | 1.0497 | ↑ | 0.8899 |
| 63 | ERO1L | <0.0001 | 1.1191 | ↑ | 0.8841 | 152 | SPHK1 | <0.0001 | 1.3345 | ↑ | 0.8236 |
| 64 | FADD | <0.0001 | 1.0358 | ↑ | 0.7318 | 153 | SPNS1 | <0.0001 | 1.0632 | ↑ | 0.8963 |
| 65 | FAM48A | <0.0001 | 1.0227 | ↑ | 0.6941 | 154 | SQSTM1 | <0.0001 | 1.0458 | ↑ | 0.8362 |
| 66 | FAS | <0.0001 | 1.1497 | ↑ | 0.9039 | 155 | ST13 | 0.0084 | 0.9883 | ↓ | 0.6011 |
| 67 | FKBP1A | <0.0001 | 1.0562 | ↑ | 0.8980 | 156 | STK11 | 0.0042 | 1.0163 | ↑ | 0.6323 |
| 68 | FKBP1B | <0.0001 | 0.8328 | ↓ | 0.8404 | 157 | TBK1 | <0.0001 | 1.0321 | ↑ | 0.8023 |
| 69 | FOS | <0.0001 | 0.9269 | ↓ | 0.6763 | 158 | TM9SF1 | <0.0001 | 0.9773 | ↓ | 0.7053 |
| 70 | FOXO1 | <0.0001 | 0.9188 | ↓ | 0.8952 | 159 | TMEM49 | <0.0001 | 1.0595 | ↑ | 0.8423 |
| 71 | GAA | <0.0001 | 1.0272 | ↑ | 0.6874 | 160 | TMEM74 | <0.0001 | 0.4482 | ↓ | 0.8693 |
| 72 | GABARAPL1 | <0.0001 | 0.8589 | ↓ | 0.9603 | 161 | TNFSF10 | <0.0001 | 1.0454 | ↑ | 0.6990 |
| 73 | GABARAPL2 | <0.0001 | 0.9573 | ↓ | 0.8632 | 162 | TP53 | <0.0001 | 1.0563 | ↑ | 0.8332 |
| 74 | GAPDH | <0.0001 | 1.0954 | ↑ | 0.9688 | 163 | TP63 | <0.0001 | 0.8318 | ↓ | 0.6681 |
| 75 | GNB2L1 | <0.0001 | 1.0845 | ↑ | 0.9551 | 164 | TP73 | <0.0001 | 2.9322 | ↑ | 0.9197 |
| 76 | GRID1 | <0.0001 | 1.2651 | ↑ | 0.7553 | 165 | TSC2 | 0.0024 | 1.0175 | ↑ | 0.6893 |
| 77 | GRID2 | <0.0001 | 0.3417 | ↓ | 0.8413 | 166 | TUSC1 | <0.0001 | 1.0615 | ↑ | 0.7884 |
| 78 | HDAC1 | <0.0001 | 0.9776 | ↓ | 0.6401 | 167 | ULK1 | <0.0001 | 1.0457 | ↑ | 0.7231 |
| 79 | HDAC6 | 0.0008 | 0.9782 | ↓ | 0.6115 | 168 | ULK2 | 0.0472 | 0.9880 | ↓ | 0.5615 |
| 80 | HGS | <0.0001 | 1.0329 | ↑ | 0.7606 | 169 | ULK3 | 0.0009 | 1.0325 | ↑ | 0.6396 |
| 81 | HIF1A | <0.0001 | 0.9076 | ↓ | 0.8887 | 170 | UVRAG | 0.0052 | 1.0131 | ↑ | 0.6744 |
| 82 | HSPA8 | <0.0001 | 0.9499 | ↓ | 0.8335 | 171 | VAMP3 | 0.0015 | 1.0124 | ↑ | 0.6783 |
| 83 | HSPB8 | <0.0001 | 1.2442 | ↑ | 0.9464 | 172 | VAMP7 | <0.0001 | 0.9431 | ↓ | 0.9398 |
| 84 | IFNG | <0.0001 | 5.1930 | ↑ | 0.9038 | 173 | VEGFA | <0.0001 | 1.2856 | ↑ | 0.9643 |
| 85 | IKBKB | 0.0028 | 1.0190 | ↑ | 0.6452 | 174 | WDFY3 | <0.0001 | 0.9661 | ↓ | 0.7092 |
| 86 | IKBKE | <0.0001 | 1.0815 | ↑ | 0.6754 | 175 | WDR45 | 0.0014 | 1.0200 | ↑ | 0.6258 |
| 87 | IL24 | <0.0001 | 1.5765 | ↑ | 0.7258 | 176 | WIPI2 | 0.0027 | 0.9851 | ↓ | 0.7087 |
| 88 | ITGA3 | 0.0002 | 1.0362 | ↑ | 0.6713 | 177 | ZFYVE1 | <0.0001 | 0.9516 | ↑ | 0.8212 |
| 89 | ITGB1 | <0.0001 | 1.0405 | ↑ | 0.7970 |  |  |  |  |  |  |
