# Supplementary material for: Autophagy-related gene P4HB: a novel diagnosis and prognosis marker for kidney renal clear cell carcinoma
Source: Aging (Albany NY). 2020 Jan 30;12(2):1828–42. doi: 10.18632/aging.102715 (PMC7053637; doi:10.18632/aging.102715)
Supplement: Supplementary Table 3 [file aging-12-102715-s002..docx]

Supplementary Table 3. The mRNA expression of *P4HB*, *GABARAPL1* and *Casp4* in KIRC through Oncomine.

| **Gene** | **Dataset** | **Normal (Cases)** | **Tumor (Cases)** | **Fold change** | **t-Test** | ***p*-value** |
| --- | --- | --- | --- | --- | --- | --- |
| *Casp4* | Beroukhim | Renal Cortex (10)/Renal Tissue (1) | KIRC (27) | 2.697 | 7.689 | 6.81E-9 |
| *P4HB* | Beroukhim | Renal Cortex (10)/Renal Tissue (1) | KIRC (27) | 2.012 | 8.217 | 2.98E-8 |
| *GABARAPL1* | Beroukhim | Renal Cortex (10)/Renal Tissue (1) | KIRC (27) | -3.724 | -10.707 | 8.80E-13 |
